# Immune complex-induced haptokinesis in human non-classical monocytes

**DOI:** 10.3389/fimmu.2023.1078241

**Published:** 2023-03-01

**Authors:** Sophie L. Preuß, Stephanie Oehrl, Hao Zhang, Thomas Döbel, Ulrike Engel, Jennifer L. Young, Joachim P. Spatz, Knut Schäkel

**Affiliations:** ^1^ Department of Dermatology, University Hospital Heidelberg, Heidelberg, Germany; ^2^ Nikon Imaging Center, Heidelberg University, Heidelberg, Germany; ^3^ Department of Cellular Biophysics, Max Planck Institute for Medical Research, Heidelberg, Germany; ^4^ Mechanobiology Institute, National University of Singapore, Singapore, Singapore; ^5^ Biomedical Engineering Department, National University of Singapore, Singapore, Singapore; ^6^ Department of Biophysical Chemistry, Heidelberg University, Heidelberg, Germany

**Keywords:** non-classical monocytes, 6-sulfo LacNAc^+^ monocytes, immune complexes, haptokinesis, Fc gamma receptor, CD16, ADAM17, cell migration

## Abstract

Formation and deposition of immune complexes (ICs) are hallmarks of various autoimmune diseases. Detection of ICs by IC receptors on leukocytes induces downstream signaling and shapes the local immune response. In many cases the pathological relevance of ICs is not well understood. We here show that ICs induce a distinct migratory response, i.e. haptokinesis in 6-sulfo LacNAc^+^ monocytes (slanMo) and in non-classical monocytes (ncMo) but not in intermediate (imMo) and classical monocytes (cMo). Using live imaging combined with automated cell tracking, we show that the main features of IC-dependent haptokinesis are elongation of the cell body, actin polarization at the leading edge, and highly directional migration. We find that CD16-dependent signaling mediates haptokinesis as blocking of CD16 or blocking SYK-signaling inhibited the migratory response. The activity of the metalloproteinase ADAM17 also modifies IC-dependent haptokinesis, likely at least partially *via* cleavage of CD16. Furthermore, using matrices with defined ligand spacing, we show that ligand density impacts the magnitude of the migratory response. Taken together, we have demonstrated that ICs induce a specific migratory response in ncMo but not in other monocyte subsets. Therefore, our work lays the groundwork for the investigation of IC-dependent haptokinesis in ncMo as a potential pathomechanism in IC-mediated autoimmune diseases.

## 1 Introduction

Generation and deposition of immune complexes (ICs) play an important role in both health and disease. When the antigen-binding Fab domains of immunoglobulins engage microbial, viral, self- or tumor antigens either soluble or surface-bound ICs are formed. Binding of antigen to the Fab domain of immunoglobulins increases the affinity of their Fc portion to Fc receptors that are expressed on various leukocytes. This increased affinity to and the cross-linking of Fc receptors on leukocytes allow the immune system to mount a cellular response to the presence of the antigen (1). Under physiological conditions, IC formation, clearance, and IC-dependent effector functions are required for adequate immune responses. Direct effector functions that are induced by the presence of ICs include neutralization and complement activation while powerful cellular responses include antibody-dependent cell-mediated cytotoxicity (ADCC) (2–4). However, excessive accumulation and inappropriate clearance of ICs can lead to inflammation and tissue injury. Consequently, the dysregulation of IC formation and clearance is associated with autoimmune diseases like systemic lupus erythematosus, rheumatic diseases, and different forms of vasculitis (5–8).

We have previously shown that 6-sulfo LacNAc^+^ (slan) monocytes (slanMo) can be recruited from the flow and activated by ICs that were deposited in the vascular bed. Recruitment was mediated by CD16 (FcγRIIIA), which has pronounced capacity for binding and handling of ICs (9, 10). Based on their differential expression of CD14 and CD16, human monocytes are separated into classical (cMo, CD14^+^ CD16^-^), non-classical (ncMo, CD14^-^ CD16^+^) and intermediate (imMo, CD14^+^ CD16^+^) monocytes (11–13). SlanMo are a subpopulation of ncMo with a particularly low expression of CD14. The defining marker slan is a carbohydrate modification of the cell surface protein PSGL-1 and is recognized by the mAb M-DC8 (14, 15). About half of the ncMo show an expression of slan.

After exiting the bone marrow, monocytes of the peripheral blood exhibit diverse and subset-specific functional properties. Among them is the rapid recruitment to sites of inflammation where monocytes exert effector functions that can both supplement macrophages and dendritic cells (16). Human ncMo were shown to be pro-inflammatory in a number of chronic inflammatory diseases. For example, we have shown that slanMo accumulate in class III lupus nephritis where they act proinflammatory through production of TNFα (10). Furthermore, it was recently shown, that ncMo are the first cells to be recruited during nephrotoxic nephritis (17). Additionally, proinflammatory effects of human ncMo were described in psoriasis, atopic dermatitis, lupus erythematosus, multiple sclerosis and Crohn’s disease (18–22).

As compared to Ly6C^hi^CX3CR1^int^ cMo, murine ncMo are Ly6C^lo^CX3CR1^hi^ (23, 24). It is currently assumed that during homeostasis only a fraction of murine ncMo is extravascular (25). It was shown that they patrol the luminal side of the vascular endothelium, where they scavenge microparticles and survey the integrity of endothelial cells (25, 26). Similarly, human ncMo but not imMo that were transferred into mice showed patrolling behavior (23). However, these studies also demonstrated that quick extravasation may occur during inflammation. Additionally, ncMo were shown to take up cellular debris, clear nucleic acids, and phagocytose tumor material (23, 27).

To allow patrolling behavior, cell adhesion, reorganization of the cytoskeleton, cell protrusion, and cell detachment must be tightly regulated. However, the underlying mechanisms that govern patrolling behavior of ncMo are incompletely understood. So far, it is known that ß_2-_integrins LFA-1 and MAC-1 as well as ICAM-1 and CD36 play an important role during patrolling activity (25, 28–31). Additionally, among others, the chemokine CX3CL1 (32), TLR-agonists like Resiquimod (R484) (29), CYR61/CCN1 (30), TNF-α (28), oxidized LDL (31) and tumor cells (27) were reported to alter the kinetics of the patrolling behavior.

Based on the observation that immobilized ICs can recruit ncMo from the flow we asked whether deposited ICs may have an additional impact on subsequent migratory behavior. Our work reveals that surface-bound ICs induce haptokinesis in slanMo. We show that IC-dependent haptokinesis depends on CD16 and is modulated by the activity of ADAM17. Therefore, our work strongly suggests that ICs that are deposited in the vascular bed and in tissues are an important factor altering the migratory behavior of ncMo.

## 2 Materials and methods

### 2.1 Cell isolation

PBMCs from buffy coats were isolated using density gradient centrifugation over Ficoll (Biochrom, Cambridge, United Kingdom). Isolation of slanMo was carried out by magnetic cell separation using an AutoMACS Pro Separator (Miltenyi Biotec, Bergisch Gladbach, Germany) as described before (33). Briefly, PBMCs, which were isolated by density gradient centrifugation were counted and centrifuged at 4°C for 10 min. Then, 1ml of 1:100 diluted DD2 hybridoma supernatant per 1x10^8^ PBMCs was added and incubated for 15 min at 4°C. Next, washing with PBS/2mM EDTA was performed, and cells were centrifuged at 4°C for 10 min. Cells were resuspended in 400 μL MACS buffer per 1x10^8^ PBMCs. Then, 20 μL per 1x10^8^ PBMCs anti-mouse IgM microbeads were added and incubated for 15 min at 4°C. After that, cells were washed, centrifuged, and resuspended in 400 μl MACS buffer per 1x10^8^ PBMCs and slanMo were positively selected using AutoMACS Pro Seperator. To analyze the purity of isolated cells, they were analyzed by FACS using a BD FACSCanto (BD Biosciences, Heidelberg, Germany). Therefore, cells were stained with antibodies specific for CD16, CD3, propidium iodide and slan. Only cell suspensions that had a purity of more than 95% were used.

Isolating the subpopulation of slanMo allows a mAb-directed cell isolation of ncMo in short time and few steps. Thus, in most experiments slanMo were analyzed as a subpopulation of ncMo as experiments revealed that slan^+^ and slan^-^ ncMo share the property of an IC-induced haptokinesis.

For direct comparison of monocytes subsets, purification of imMo, ncMo, slan^+^ and slan^-^ monocytes was performed in a two-step process (**Figure S1**). First, CD16-expressing PBMCs were isolated by preincubation with a non-blocking monoclonal antibody against CD16 (clone DJ130C, GeneTex, Irvine, California), followed by a PE-labeled anti-mouse IgG secondary antibody (Miltenyi Biotec, Bergisch Gladbach, Germany), followed by anti-PE microbeads (Miltenyi Biotec, Bergisch Gladbach, Germany) and subsequent elution *via* MACS-Separation. A series of experiments was performed with slanMo to verify that clone DJ130c does not impact haptokinesis (**Figure S2**). In a second step, eluted CD16-expressing cells were labeled with fluorochrome-conjugated antibodies against CD16, CD14, HLA-DR, CD56 and slan. Monocyte subsets were then gated and sorted using a BD FACS Melody Cell Sorter (BD Biosciences, Heidelberg, Germany). CMo were purified from PBMCs using the Classical Monocyte Isolation Kit (Miltenyi Biotec, Bergisch Gladbach, Germany) according to the manufacturer’s instructions.

### 2.2 Preparation of immune complexes

ICs were prepared as described before (9). In short, FITC-labeled human serum albumin (HSA-FITC, Abcam, Cambridge, United Kingdom) at a stock concentration of 1 mg/ml was used as antigen. Polyclonal rabbit IgG anti-FITC (Abcam, Cambridge, United Kingdom) at a stock concentration of 1 mg/ml was used as antibody component of the ICs. HSA-FITC and anti-FITC were mixed at a ratio of 1:4 and incubated for one hour at 4°C. Stock solutions were stored at 4°C.

### 2.3 Haptokinesis assay

Haptokinesis assays with surface-bound ICs were performed in µ-slides chemotaxis IV 0.4 (IBIDI, Martinsried, Germany). For coating of the slides with ICs, the IC stock was diluted 1:120 in PBS. Each well of the uncoated µ-slides was then loaded with 100μl of diluted ICs and incubated overnight at 4°C. Control samples were a 1:100 dilution of HSA-FITC, a 1:200 dilution of RGD peptides (Sigma-Aldrich, St. Louis, Missouri), and PBS. Further control samples were IgE-ICs and R848 treatment (**Figure S3**). Before experiments started all slides were rinsed three times with PBS.

To enable tracking of cells, monocyte subsets were incubated with 1 μM CFSE (Biolegend, San Diego, California) for five min at RT. Cells were then adjusted to a concentration of 1x10^6^ cells per ml and 100 μl of the cell suspension per well was carefully applied to the slides. For each independent experiment cells of the same donor were applied at the same time to the different wells. After transfer of the slides to a stage top incubator equilibrated at 37°C, cells were imaged for 90 min using the Nikon Ti2 microscope (Nikon, Tokio, Japan). For each well, one to three fields of view were imaged every 90 seconds. After image acquisition, automated cell tracking was performed using the NIS-Elements 5.20.02 software (Nikon, Tokio, Japan). Tracks were manually excluded if they were clearly the result of incorrect tracking, for example inclusion of two cells in one track.

In some experiments cells were pre-treated immediately before the experiments with blocking antibodies or reagents. Blocking of CD16 was achieved by pre-incubation of slanMo with 100 μg/ml anti-human CD16 (clone 3G8, BD Biosciences, Heidelberg, Germany) or mouse IgG1 (MOPC21) (Biolegend, San Diego, California) at the same concentration as isotype antibody for 15 min at 4°C. Blocking of ADAM17 was achieved by pre-incubation of slanMo with 50 nM anti-human ADAM17 (clone 3D1(A12), Abcam, Cambridge, United Kingdom) or human IgG (31154) (Thermo Fisher, Waltham, Massachusetts) at the same concentration as isotype antibody for 15 min at 4°C. SYK-inhibition was achieved by preincubation of slanMo with a SYK inhibitor (4 μM) (BAY61-3606) (Enzo LifeSciences, Lörrach, Germany) for 15 min at 4°C. Toxicity of BAY61-3606 was excluded by performing a toxicity assay (**Figure S4**).

Haptokinesis assays on endothelial cells were performed in 0.8 mm µ slides I Luer (IBIDI, Martinsried, Germany). Prior to seeding, slides were coated with 5 µg/cm^2^ fibronectin (Merck, Kenilworth, New Jersey) for one hour at RT. Excess liquid was then removed and slides were rinsed with PBS. 2.5x10^5^ human umbilical vein endothelial cells (HUVECs, Promocell, Heidelberg Germany) in human endothelial cell growth medium (Promocell, Heidelberg, Germany) were then added to the slides and cultured overnight to form a monolayer. Immediately before the experiment, the medium was replaced with fresh medium either with or without 12.5 µg/ml rabbit anti-human CD105 (Abcam, Cambridge, United Kingdom) followed by incubation for 10 min at 37°C. CD105 is highly expressed on the cell surface of endothelial cells. Previous experiments of our group did show a homogeneous expression on HUVECs and a high affinity of rabbit-anti human CD105 mAb. In addition, we could demonstrate the capture of slanMo from the flow employing this experimental setting (10). Purified slanMo were resuspended at a concentration of 1x10^6^ cells/ml in x-vivo (Lonza Group, Basel, Switzerland) and 250 µl of the cell suspension were added to the HUVEC cultures. Slides were imaged for 90 min at 37°C using an EVOS M7000 imaging system (Thermo Fisher, Waltham, Massachusetts). Photos were taken every 60 sec and movement of slanMo was tracked using the Celleste Image Analysis Software (Thermo Fisher, Waltham, Massachusetts).

### 2.4 Immunofluorescence

Ibidi μ-slides were coated with ICs as described above. SlanMo were then added to the slides and incubated for 20 min at 37°C. After incubation, cells were fixed with ice cold PFA (3-4%) in PBS for 20 min. For F-Actin staining cells were permeabilized with 0.1% Saponin followed by incubation with 25 μl/ml of Alexa Fluor^®^ 647 Phalloidin (Thermo Fisher, Waltham, Massachusetts) for 40 min at RT. For DAPI staining cells were incubated with a 1:20.000 dilution of DAPI (Sigma-Aldrich, St. Louis, Missouri) and rinsed with distilled water. Image acquisition was performed with the Nikon A1R confocal microscope and the Nikon Ti2 microscope (Nikon, Tokio, Japan).

### 2.5 Gold nanoparticle slides

Gold nanoparticle slides were prepared by block copolymer micellar nanolithography and regions outside of the nanoparticles were poly(ethylene glycol) (PEG) passivated in collaboration with the group of Joachim Spatz at the Max Plank Institute for Medical Research (Heidelberg) as previously described (34–36). For functionalization, 4.6 μl Traut’s reagent (14 mM) (Thermo Fisher, Waltham, Massachusetts) were added to 100 μl (1 mg/ml) IgG3 (Biolegend, San Diego, California) followed by incubation for one hour at RT. The thiolated antibody was separated from Traut’s reagent with a Zeba Spin Desalting Column (Thermo Fisher, Waltham, Massachusetts). 80 μl of the thiolated antibody was applied to a sheet of parafilm. The gold nanoparticle coverslips were placed upside down on the antibody solution and incubated for two hours in the dark. After incubation, the coverslips were rinsed carefully with PBS.

### 2.6 Statistical analysis

Wilcoxon, Mann-Whitney U test, Friedmann and Kurskal-Wallis-Test combined with Dunn’s *post hoc* test were used for statistical analysis as indicated. Levels of significance were labeled as follows: *p < 0.1, **p < 0.01, ***p < 0.001, ****p < 0.0001. Directionality was assessed by dividing the euclidian distance (shortest distance between initial and final position in a straight line) and the accumulated distance of the total cell migration path. Analyses were performed and graphs were designed using GraphPad Prism (GraphPad Software, Inc., San Diego, California) and BioRender (Toronto, Canada). Polar Plots were created using the Chemotaxis and Migration Tool 2.0 (IBIDI, Martinsried, Germany) and Fiji.

## 3 Results

### 3.1 Endothelial cell-bound antibodies and surface-bound ICs induce a directional haptokinetic response in slanMo

**Figure 1:**
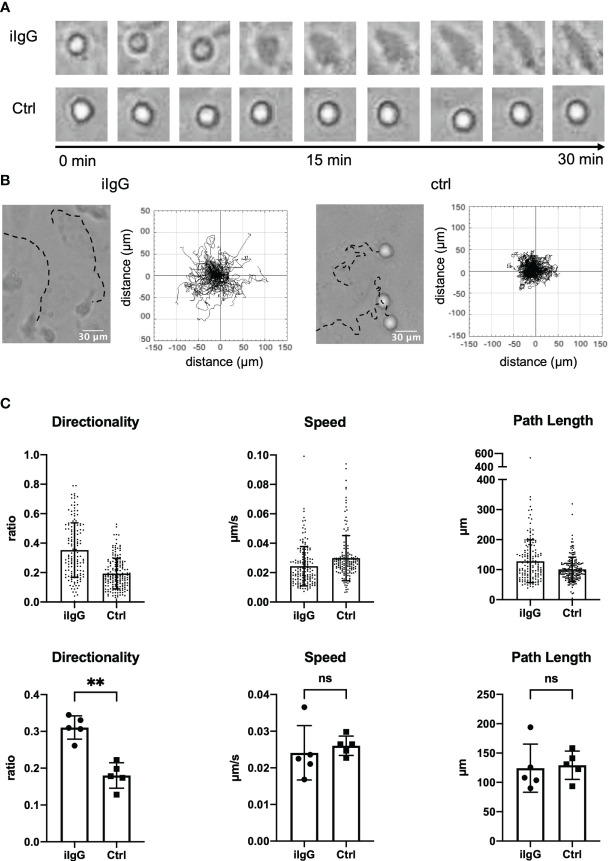
Antibodies deposited on endothelial cells induce a specific haptokinetic response in slanMo. **(A)** A monolayer of human umbilical vein endothelial cells (HUVECs) was incubated for 10 min with an antibody targeting CD105. Immediately after incubation a cell suspension of slanMo was applied to the cell culture. The upper row of images shows attachment, spreading, and elongation of slanMo on antibody-targeted endothelial cells. The lower row of images shows retention of spherical cell shape on non-targeted endothelial cells. N = 5. Representative cells and images from one experiment are shown **(B)** Representative tracks of the migratory response of slanMo on antibody-targeted endothelial cells (left) and non-targeted endothelial cells (right). Trajectory plots of slanMo on non-targeted and antibody-targeted endothelial cells. N = 5. Representative results from one experiment are shown. **(C)** Quantification of the haptokinetic response of slanMo on antibody-targeted endothelial cells and non-targeted cells. Directionality is a measure of the straightness of cell migration. Above: Each dot represents a tracked cell of one representative experiment. Below: Each dot represents the mean of all tracked cells from one experiment. N = 5. Error bars show mean ± SD, **p < 0.01. ns, not significant. Wilcoxon test.

We have previously shown that ICs recruit slanMo from the blood flow and lead to activation of the cells, which suggested that slanMo may be a proinflammatory cell type in human lupus nephritis (10). To further understand how surface-bound ICs may affect the behavior of slanMo, we first set up an assay, in which slanMo interacted with a monolayer of human umbilical vein endothelial cells (HUVECs) that were targeted with antibodies against CD105 immediately before the experiment. On antibody-targeted endothelial cells, slanMo attached, then spread, and subsequently initiated a specific directional but not collectively oriented migratory response, that is haptokinesis. No such response could be observed on endothelial cells that were not antibody-treated (**Figure 1A**). A striking characteristic during initiation of IC-induced haptokinesis is the specific elongated phenotype that could be observed in the majority of slanMo while a rounder more spherical phenotype was retained on non-targeted endothelial cells (**Figure 1A**). The kinetics of slanMo on antibody-targeted endothelial cells was clearly different in that the paths of attached slanMo displayed a much higher directionality as compared to controls while the overall distance covered and the speed of the cells were not significantly different (**Figures 1B, C**).

**Figure 2:**
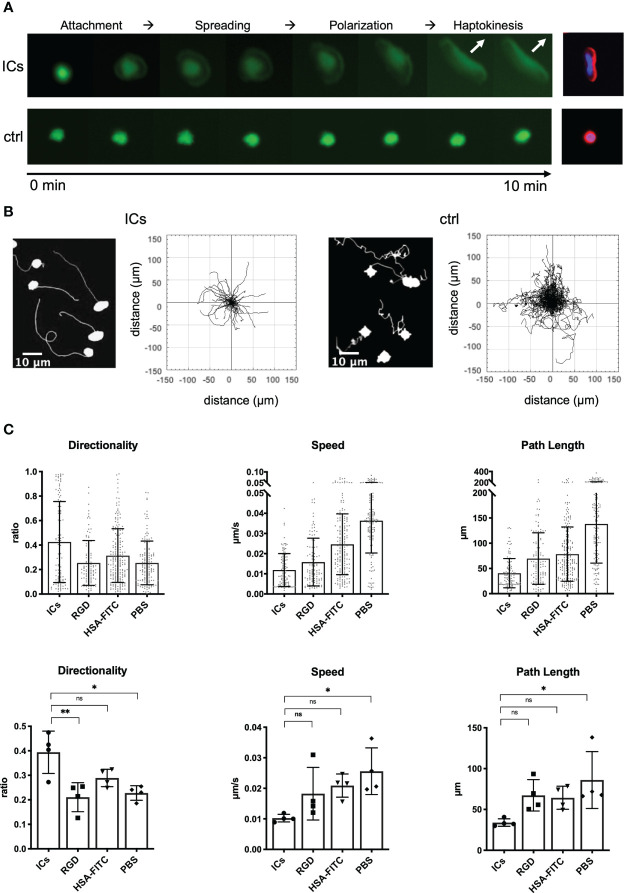
Surface-coated ICs are a model to study the haptokinetic response of slanMo. **(A)** Images of the haptokinetic response of CFSE-labeled slanMo on an IC-coated surface over a period of 10 min. Control cells were incubated in the presence of an HSA-FITC-coated surface. Different steps of the haptokinetic response are labeled based on apparent morphological and kinetic changes. The images on the right show actin polarization of haptokinetic cells after 10 min (red: F-actin, blue: DAPI). Representative images of more than 10 experiments are shown. **(B)** Left: Tracks of slanMo on IC-coated slides and PBS-treated slides over the incubation time of 30 min. Right: Trajectory plots of slanMo based on 90 min of automated cell tracking. **(C)** Quantification of directionality, speed and path length of slanMo on surfaces coated with ICs, RGD peptides and HSA-FITC or on PBS-treated surfaces. Above: Each dot represents a tracked cell of one representative experiment. Below: Each dot represents the mean of all tracked cells from one experiment. N = 4. Error bars show mean ± SD, *p < 0.1, **p < 0.01. ns, not significant. Friedmann Test and Dunn’s *post hoc* test.

To be able to systematically examine this migratory response under more defined conditions, we now chose an experimental setup with plastic surface-bound ICs and high cell density (see Methods for details). SlanMo interacting with surface-bound ICs displayed the same haptokinetic response as slanMo interacting with antibody-targeted endothelial cells – after attachment, slanMo spread, elongated, and then started to migrate within less than 10 min (**Figure 2A**). F-actin staining showed that spreading and elongation of the cells corresponded to actin polarization. The majority of slanMo on ICs displayed polarized actin on the leading edge while on HSA-coated control slides F-actin remained homogeneously distributed in slanMo (**Figure 2A**). We also repeated these experiments with surface-coated RGD peptides, the binding epitopes of integrins. However, like on HSA-coated slides and PBS-treated control slides slanMo did not display an enhanced haptokinetic response. Also, IgE-ICs and stimulation with R848 treatment did not induce haptokinesis (**Figure S3**). Under the more defined conditions of surface-bound ICs it also became apparent that the migration on ICs is slower and more directional as compared to the controls (**Figures 2B, C**). Taken together, these data show that cell surface-bound antibodies and surface-coated ICs induce a directional, IC-specific haptokinetic response in slanMo.

### 3.2 IC-induced haptokinesis is limited to ncMo, including slanMo

**Figure 3:**
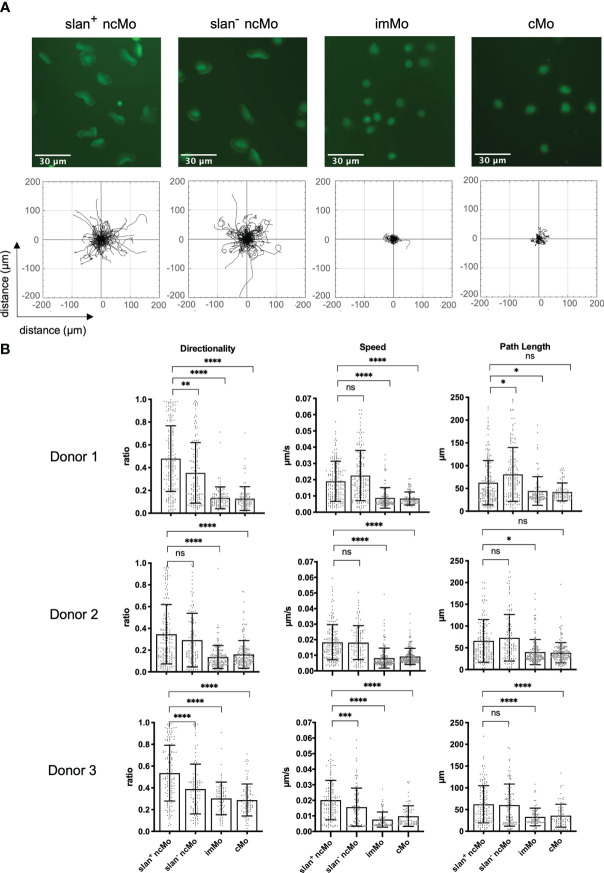
IC-dependent haptokinesis is monocyte subset-specific. **(A)** Upper panels: Morphology of monocyte subsets after incubation for 90 min on an IC-coated surface. Lower panels: Trajectory plots of monocyte subsets based on 90 min of automated cell tracking. Representative data of one experiment are shown. **(B)** Quantification of directionality, speed and path length of monocyte subsets on an IC-coated surface. Each dot represents a tracked cell. All tracked cells of 3 donors are shown. N = 3. Error bars show mean ± SD, *p < 0.1, **p < 0.01, ***p<0.001, ****p < 0.0001. ns, not significant. Kruskal-Wallis test and Dunn’s *post hoc* test.

Monocytes comprise several subsets that in humans are classified based on their expression of CD14 and CD16. CMo are CD14^hi^ but lack expression of CD16. ImMo are also CD14^hi^ and additionally are CD16^lo-hi^. NcMo are CD14^mid-lo^ and CD16^hi^. As slanMo are a subset of ncMo (CD14^lo^, CD16^hi^, slan^+^) we wanted to clarify whether IC-induced haptokinesis is limited to a specific cell type or shared by other related monocytic cells. To directly compare slanMo, ncMo, imMo, and cMo we FACS- and/or MACS-sorted all four subsets from one donor and then repeated our experiments on surface-coated ICs. Interestingly, both slanMo and ncMo displayed similar morphological changes and a comparable haptokinetic response while cMo and imMo did not (**Figure 3A**, upper panels). This was reflected by greater directionality, speed, and path length of slanMo and ncMo as compared to cMo and imMo (**Figure 3B**). CMo and imMo attached to the IC-coated surface and showed spreading, but neither a locomotory response nor elongation of the cell body could be observed (**Figures 3A, B**). For cMo these findings were corroborated by studying their migratory response on IgG-coated human umbilical vein endothelial cells [HUVECs (**Figure S5**)]. Taken together our data show that among monocytes the haptokinetic response after encounter of surface-bound ICs is specific to ncMo, including slanMo.

### 3.3 Signaling *via* CD16 is required for IC-induced haptokinesis

**Figure 4:**
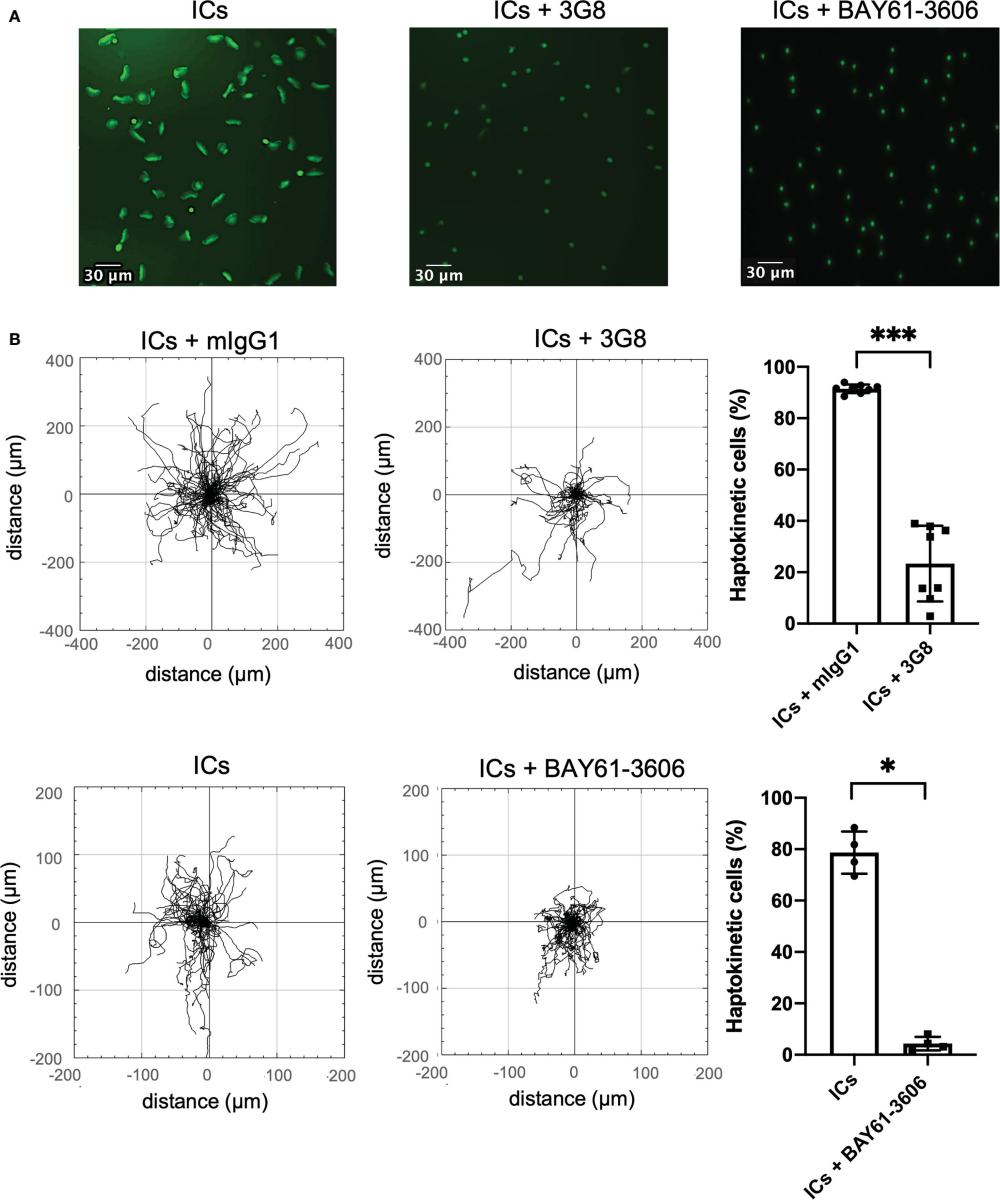
Haptokinesis of slanMo requires functional CD16. **(A)** Morphology of slanMo after 10 min of incubation on an IC-coated surface. First sample were cells that were pre-incubated with a non-blocking isotype control. Cells in the second sample were pre-treated with a blocking mAb against CD16 (clone 3G8). Cells in the third sample were incubated in the presence of a SYK inhibitor (BAY61-3606). Original magnification 20x. **(B)** Left: Trajectory plots highlighting the changes in the presence of a blocking mAb against CD16 (clone 3G8) or SYK inhibitor (BAY61-3606). Representative images and trajectory plots of one representative experiment are shown. N = 8. Upper right: Percentage of polarized haptokinetic control-treated slanMo versus slanMo that were pre-treated with a blocking mAb against CD16 (clone 3G8). Measurements were performed after 10 min of incubation. N = 8. Error bars show mean ± SD, ***p < 0.001, Wilcoxon test. Lower right: Percentage of polarized haptokinetic vehicle-treated slanMo versus slanMo that were pre-treated with SYK inhibitor (BAY61-3606). N = 4. Error bars show mean ± SD, *p < 0.1, Wilcoxon test.

Both soluble and surface-bound ICs are detected by immune cells *via* Fc receptors. We have shown previously, that CD16 on slanMo plays a pivotal role in binding of ICs and blocking of CD16 prevented cell recruitment of slanMo to antibody-coated endothelial cells from the flow (10). Therefore, we wanted to understand whether specific Fc receptors and Fc receptor signaling are required for IC-induced haptokinesis of slanMo and ncMo. Blocking of CD16 (clone 3G8) immediately before the experiment did not completely abrogate the haptokinetic response but resulted in a clear reduction of haptokinetic cells. Many slanMo with blocked CD16 displayed neither spreading nor the elongated phenotype of haptokinetic cells indicating that CD16 is important for IC-induced haptokinesis (**Figures 4A, B**). In line with this, performing the experiments in the presence of a SYK inhibitor, that inhibits signaling *via* Fc receptors, resulted in a reduction of haptokinetic cells (**Figures 4A, B**). Taken together, these results show that IC-induced haptokinesis of slanMo is mediated by CD16 and depends on active Fc receptor signaling.

### 3.5 ADAM17 is a modulator of IC-induced haptokinesis

**Figure 5:**
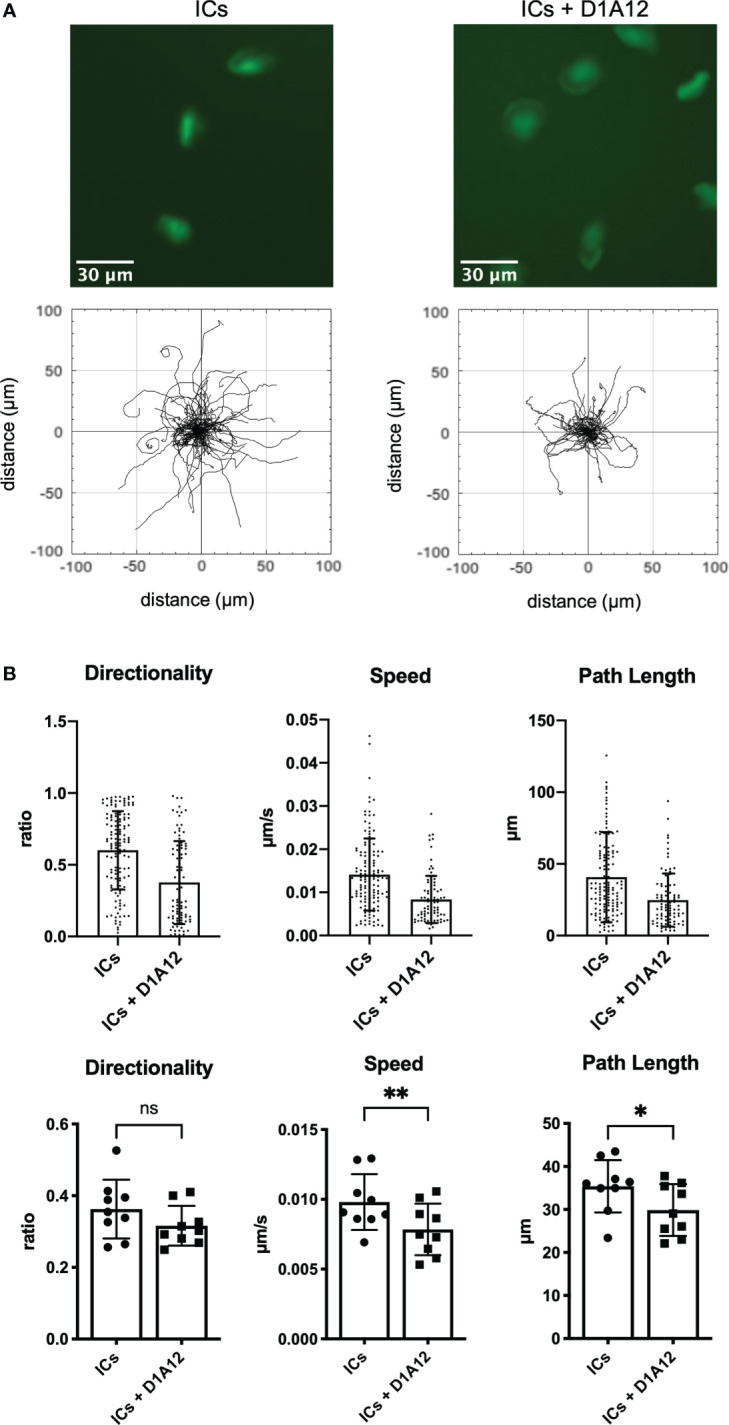
ADAM17 modulates IC-dependent haptokinesis. **(A)** Above: Morphology of slanMo after 30 min of incubation on an IC-coated surface. SlanMo of the first sample were pre-incubated with a non-blocking isotype control. SlanMo of the second sample were pre-treated with a blocking mAb against ADAM17 (clone D1A12). Original magnification 20x. Representative images of one experiment are shown. N = 9. Below: Trajectory plots after image acquisition for 90 min highlighting the changes in the presence of a blocking mAb against ADAM17 (clone D1A12). Representative trajectory plots of one experiment are shown. N = 9. **(B)** Quantification of directionality, speed, and path length of slanMo on an IC-coated surface in the presence of a blocking mAb against ADAM17 (clone D1A12). Above: Each dot represents a tracked cell of one representative experiment. Below: Each dot represents the mean of all tracked cells from one experiment. N=9. Error bars show mean ± SD, *p < 0.1, **p < 0.01. ns, not significant. Wilcoxon test.

The metalloproteinase ADAM17 cleaves the extracellular portion of CD16 and, therefore, can modulate CD16-dependent functions (9). To investigate whether ADAM17 also affects IC-dependent haptokinesis, we preincubated slanMo with a blocking antibody against ADAM17 (clone D1A12) (37) and then incubated slanMo in the presence of surface-coated ICs. After ADAM17 blocking slanMo still showed spreading and elongation comparable to unblocked control cells, indicating that this aspect of an initial haptokinetic response is independent of ADAM17 (**Figure 5A**). However, speed and path length were significantly reduced (**Figure 5B**). Therefore, ADAM17 modulates IC-induced haptokinesis of slanMo, likely at least partially *via* ectodomain shedding of CD16.

### 3.5 IC-induced haptokinesis is dependent on ligand density

**Figure 6:**
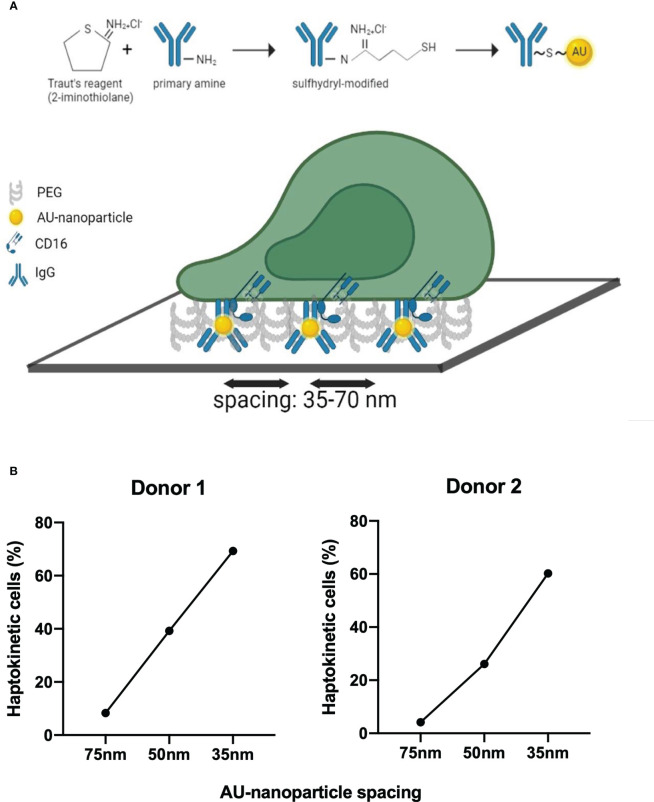
Ligand density affects IC-dependent haptokinesis **(A)** Schematic of human IgG modification and binding of human IgG to gold nanoparticles using Traut’s reagent. Defined spacing of IgG molecules is reached by preparation of the gold nanoparticle slides using block copolymer micellar nanolithography. **(B)** Analysis of the haptokinetic response of slanMo on IgG-decorated gold nanoparticle arrays with defined spacing of 35, 50, and 75 nm. Each data point represents the mean percentage of polarized haptokinetic cells after 60 min in two fields of view. Two independent experiments with different donors are shown.

ICs in tissues or cell surface-bound antibodies may be present in varying densities and the haptokinetic response of cells may differ depending on ligand density. To model different ligand densities, we made use of gold nanoparticle matrices that were created by block copolymer nanolithography (36). These matrices have defined spacings and the gold nanoparticles can be decorated with human IgG in a thiolation reaction (**Figure 6A**). We tested nanoparticle spacings of 35, 50, and 75 nm and imaged the haptokinetic response of slanMo for 180 min. We found that haptokinesis of slanMo correlated to the density of IgG-coated gold nanoparticles. The smaller the distance between gold nanoparticles the more cells spread and polarized. At 35 nm spacing more than 60% of slanMo attached, polarized, and displayed a haptokinetic response. At 75 nm spacing the percentage of haptokinetic cells was reduced to 10% (**Figure 6B**). These results show that ligand density of ICs is an important factor determining the magnitude of the haptokinetic response.

## 4 Discussion

Leukocyte migration is an essential process required for proper immune function. The arrival of immune cells to sites of inflammation is guided by multiple different soluble attractants. However, migratory behavior is also modified by surface-bound factors. The resulting motility pattern after integration of signals from immobilized factors is called haptokinesis (38). We here demonstrate that immobilized ICs serve as a haptokinetic cue for ncMo, describe their migratory behavior and define the basic requirements of this response.

The IC-mediated haptokinetic response of slanMo is characterized by highly directional paths. In the vasculature, such migration patterns were shown to be required for finding the shortest path towards an emigration site and also correlated with an increased number of extravasated cells (39). NcMo patrolling along the endothelium with deposited ICs may, therefore, use directional migration to scan a large area and to quickly arrive at sites of local endothelial cell stress. Consistent with this, ncMo were already shown to be among the first cells arriving at sites of IC deposition (10, 17). There, they may help to recruit additional cells like neutrophils to shape the local immune response (10, 29).

In our experiments we observed an initial adhesion of the cells to the IC-coated tissue culture substrate directly followed by the induction of directional migration, a reduction of the speed (~0.2 μm/s) and a reduction of the total path length of migration. The migration speed appeared slower compared to the patrolling speed of monocytes observed *in vivo* (~1,6μm/s or 100 μm/h) (25). The *in vitro* environment with a lack of shear stress from blood flow, and a rather strong IC-induced adhesion of the ncMo to the tissue culture surface in our experiments, may account for these differences in the speed of the cells. Interestingly, slanMo migrating on an endothelial monolayer were less adhesive (own observation), and in line with this, the speed nor the path length appeared altered by engagement of immobilized IgG. Moreover, it was also show that additional signals such as stimulation of monocytes with TLR agonists results in a decreased patrolling speed *in vivo* (29, 40).

IC-dependent haptokinesis required defined ligand densities with a high percentage of haptokinetic cells observed at or below 35 nm spacing. This is in line with studies on cell surface integrins, which showed that ligand spacing at the nanometer scale strongly affected migration characteristics (41). Local density of deposited ICs is, therefore, likely an important factor determining the cellular responses of ncMo. On small, cellular scales the physiological concentration of ICs is difficult to assess. By using gold nanoparticle slides with defined spacing we have provided a method to further investigate how IC density correlates with cellular responses. Whether a gradual increase in IC density has the potential to induce a unidirectional haptokinetic response of the cells may be addressed in future studies.

We identified CD16 on ncMo as an important mediator of IC-dependent haptokinesis. However, expression of CD16 alone was not sufficient as the population of CD16-positive imMo did not show the haptokinetic response. A similar difference was observed when transferring human monocyte subsets to mice, where only ncMo but not imMo were patrolling the endothelium in the steady state (23).

We have shown that blocking of ADAM17 does affect the haptokinetic response, likely at least partially *via* its ability to cleave CD16 (9). Speed and path length were reduced but the morphology of the cells was not impacted. As ADAM17 is a pleiotropic metalloproteinase, effects on other targets may also be important (42). Additionally, heterogenous expression of Fc receptors like CD32a/b and CD64, the immunoglobulin composition of the ICs, but also the expression of surface proteins that allow interaction with the cellular microenvironment will likely play a role. Irrespective of this, the relative specificity of the IC-dependent haptokinetic response is consistent with the known functions of ncMo. While cMo and partially also imMo are involved in pro-inflammatory responses and antigen presentation, an important function of ncMo in both mice and humans is patrolling the endothelium. This functionality is also accompanied by an upregulation of genes that are involved in cytoskeleton rearrangement and cell motility and, therefore, ncMo may be prone to respond quickly to local microenvironmental cues that require changes in migratory behavior or extravasation (13, 43). Indeed, increased numbers of ncMo were found in skin lesions and glomeruli of patients with systemic lupus erythematosus (10, 19). Both skin and glomeruli of lupus patients harbor disease-relevant ICs and the IC-dependent haptokinetic response of ncMo is likely to affect their migratory behavior. However, whether IC-dependent haptokinesis contributes to the pathomechanism remains to be clarified.

Overall, evidence is accumulating that the specific expression of CD16 provides ncMo with important functionality. We have shown that CD16 provides slanMo with a particularly high capacity to bind ICs (9). Additionally, CD16 is sufficient to recruit slanMo from the flow to areas of IC deposition (10). Turner-Stokes et al. showed that CD16 is important for the inflammatory function of ncMo during early nephrotoxic nephritis (17). We now demonstrate that CD16 also mediates IC-dependent haptokinesis. This process typically progressed by spreading and elongation of cells that was accompanied by F-action polarization and lamellipodium formation at the leading edge. Migration then occurred perpendicular to the longest axis of the cell. By blocking of CD16 the haptokinetic migratory response was strongly reduced and F-actin polymerization at the leading edge did not occur. Signaling downstream of Fc receptor cross-linking involves SYK, PLC, and BTK that among others activate Vav, Rac, and Rho kinases. Reorganization of the actin cytoskeleton is an important downstream effect (44, 45). Our work shows that the SYK kinase is at least partially involved in mediating IC-dependent haptokinesis. However, the details of the signaling cascade remain to be studied, although it is likely to involve other members of the known Fc receptor signaling network. Additionally, crosstalk with other surface molecules and signaling networks may occur. For example, there is evidence for an Fc receptor-integrin crosstalk that changes the affinity of integrins. Such crosstalk is likely to affect the migratory behavior of cells (46, 47). Crosslinking of Fc receptors in slanMo will also induce cytokines like TNF-α that in turn may provide autocrine stimulation to shape the local immune response (10).

Taken together, our study reveals that ICs are a so far undescribed haptokinetic stimulus for ncMo. IC engagement of CD16 and its signal transduction *via* SYK mediates haptokinesis and ADAM17 is a modulator of the migratory response. Therefore, our work provides additional evidence for ncMo being specialized to act at sites of local IC deposition and suggests further work on their role in IC-mediated diseases.

## Data availability statement

The original contributions presented in the study are included in the article/**Supplementary Material**. Further inquiries can be directed to the corresponding author.

## Ethics statement

Ethical review and approval was not required for the study on human participants in accordance with the local legislation and institutional requirements. Written informed consent for participation was not required for this study in accordance with the national legislation and the institutional requirements.

## Author contributions

SP, SO, HZ, and JY performed the experiments. SP, SO, HZ, UE, JY, JS, and KS designed the project and interpreted the data. SP, SO, TD, and KS wrote the manuscript. HZ, UE, JY, and JS critically read the manuscript. KS has final responsibility for decision to submit publication. All authors contributed to the article and approved the submitted version.
