# Supplementary material for: Immune complex-induced haptokinesis in human non-classical monocytes
Source: Front Immunol. 2023 Mar 1;14:1078241. doi: 10.3389/fimmu.2023.1078241 (PMC10014541; doi:10.3389/fimmu.2023.1078241)
Supplement: Supplementary file 1 [file DataSheet_1.docx]

Supplementary Material

## Supplementary Figures

S1


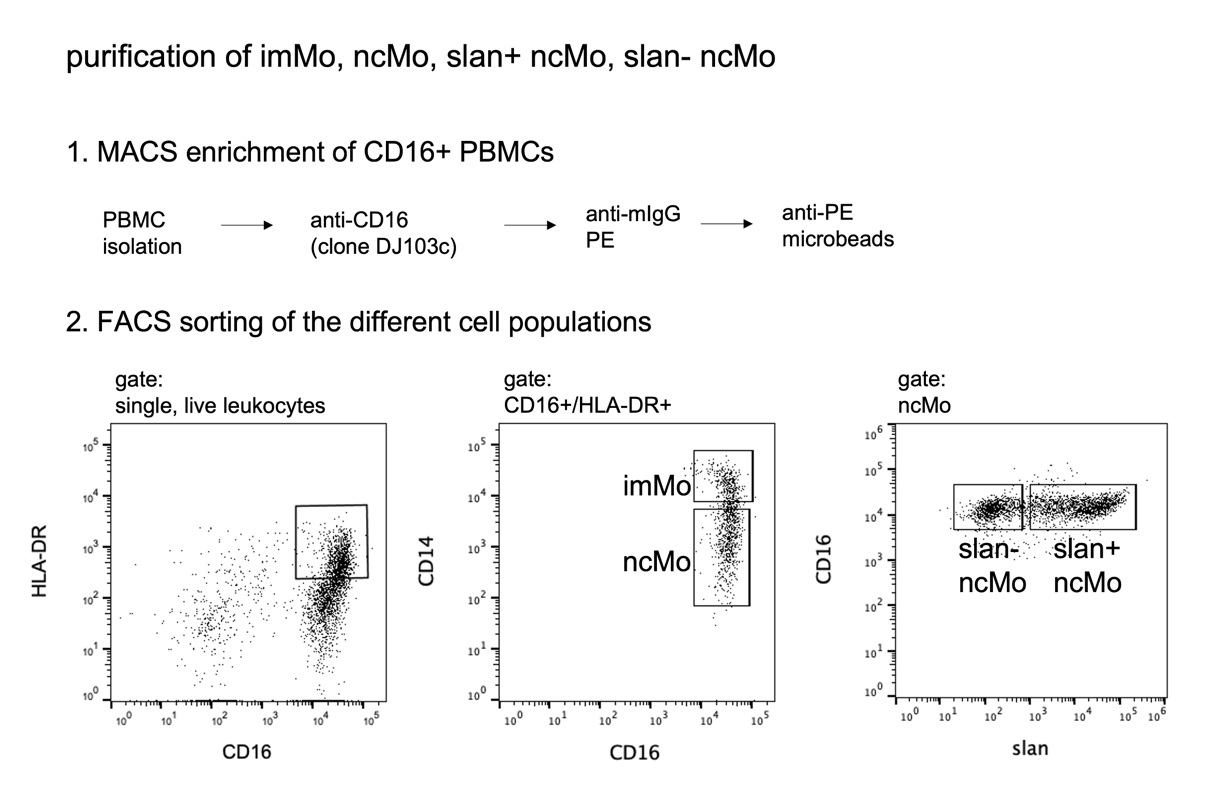


S2


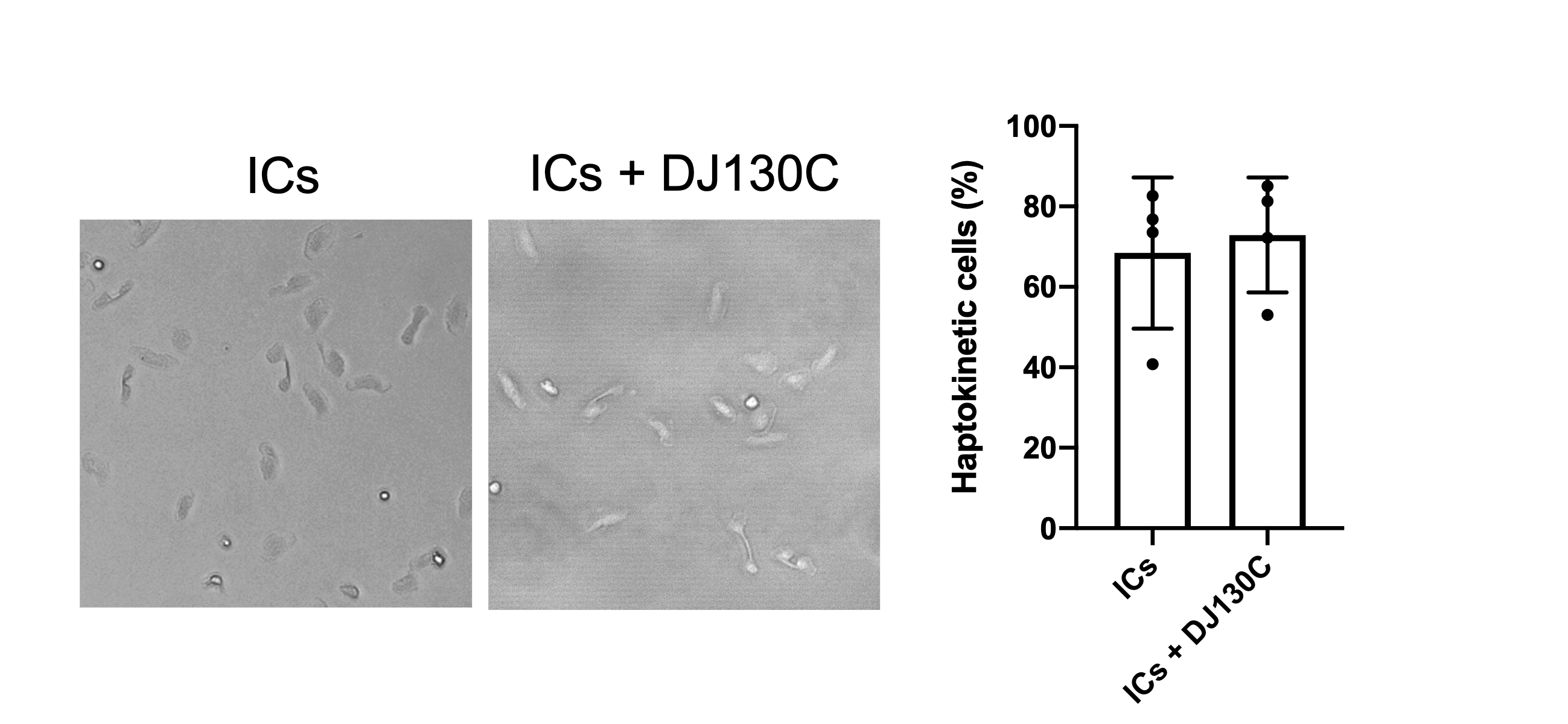


S3


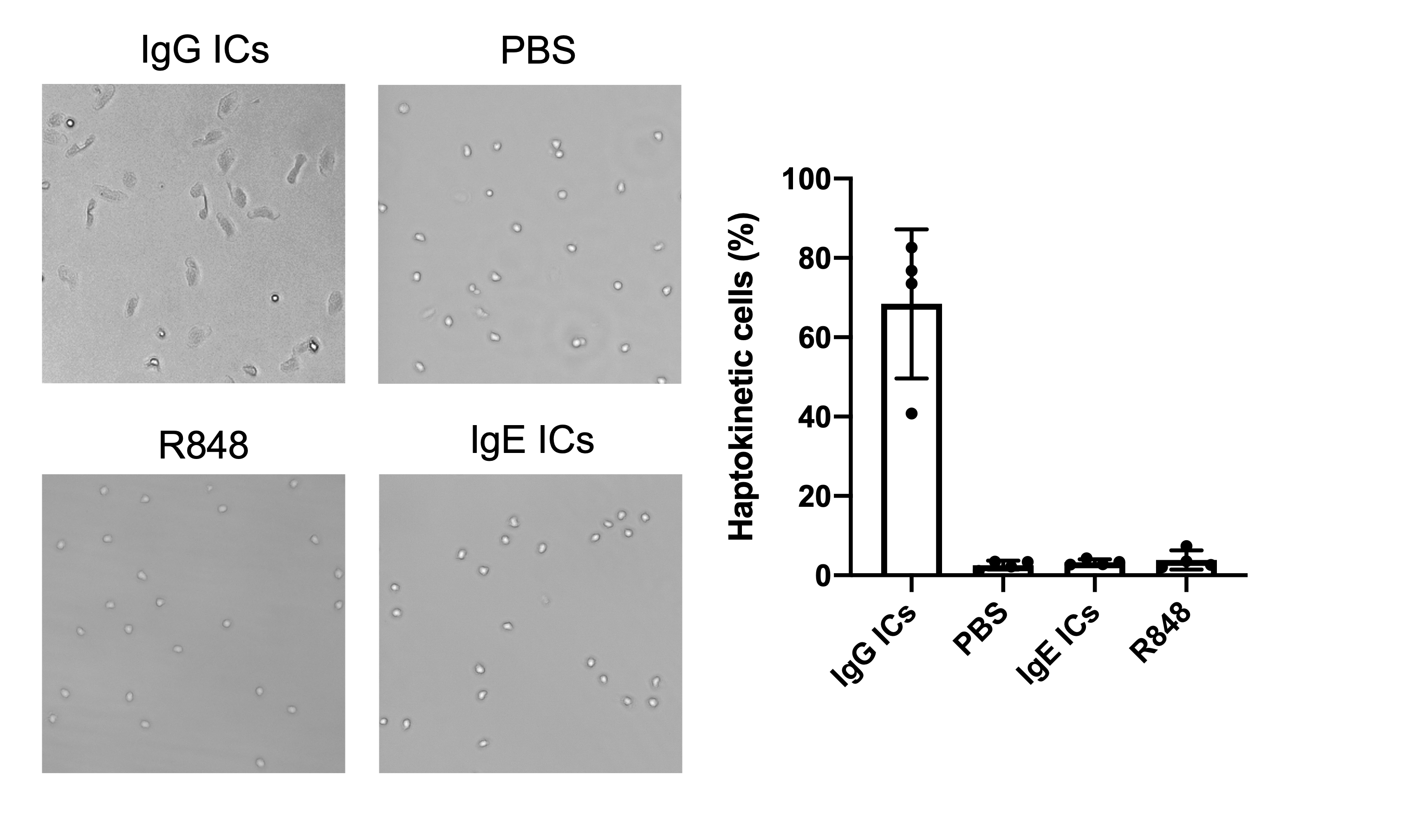


S4

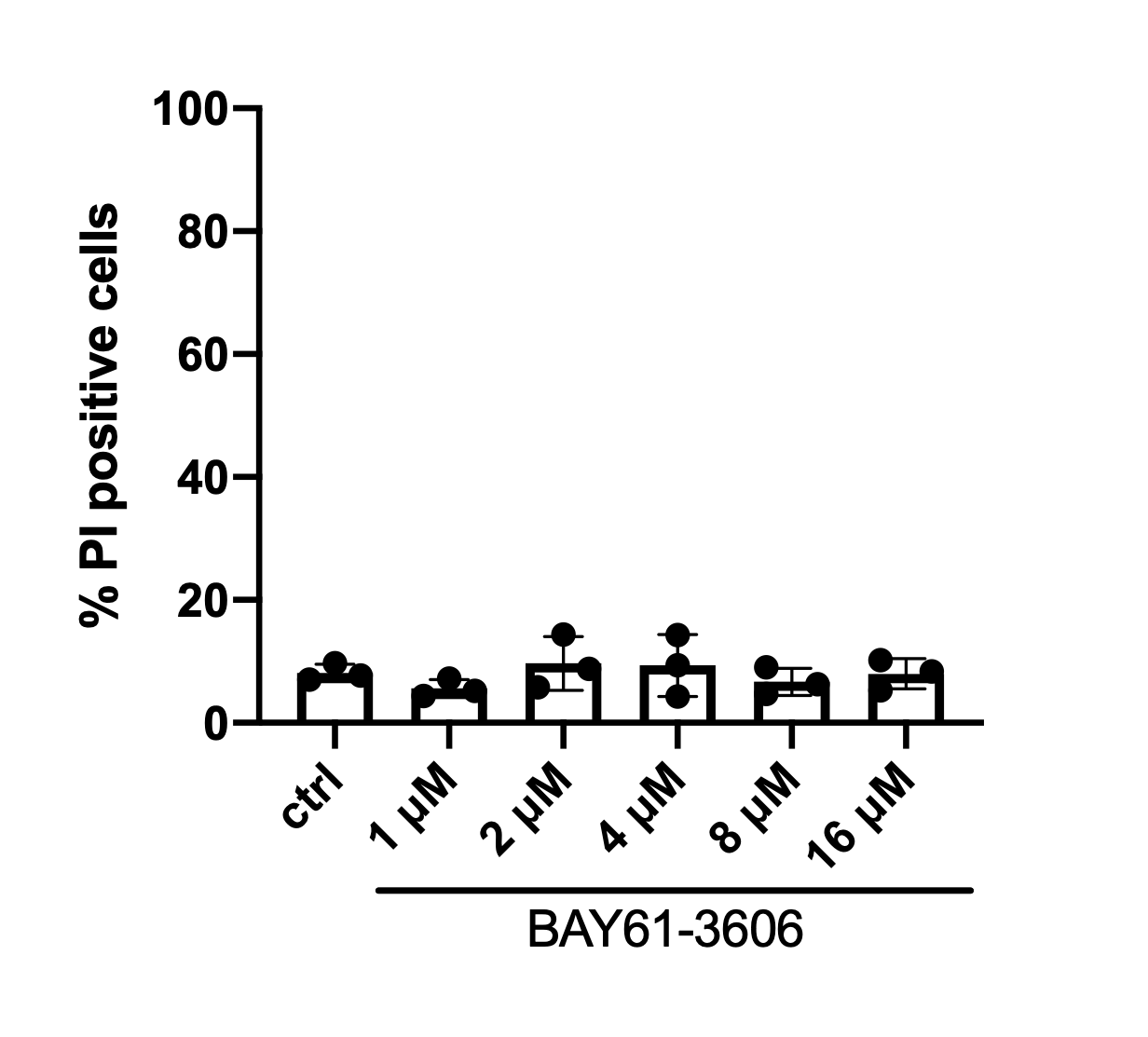


S5


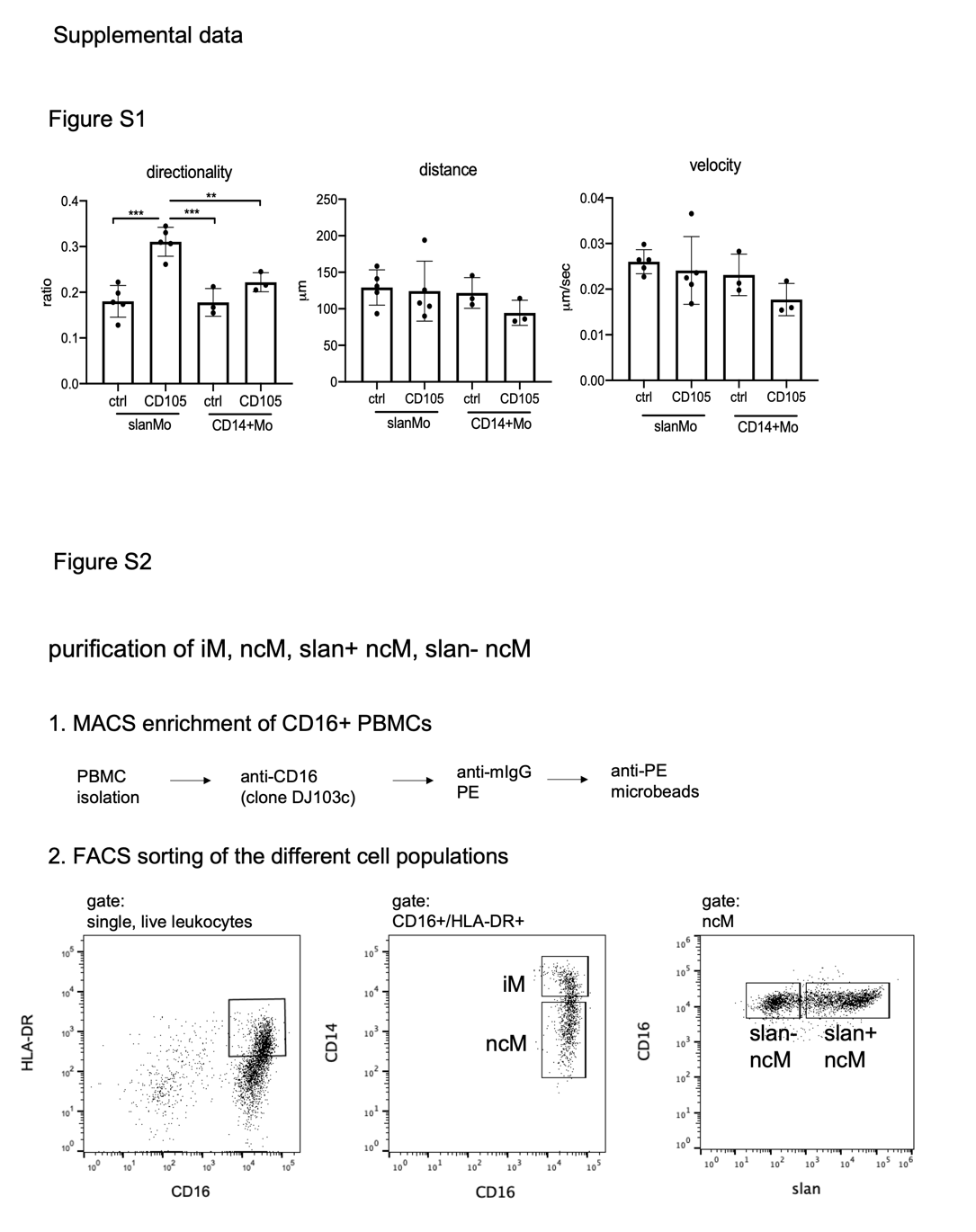


**Figure captions**

**S1 Purification of monocyte subsets.** MACS enrichment of CD16^+^ PBMCs and gating strategy for identification of the different monocyte subsets (CD16^+^ and HLA-DR^+^). Percentage of slan^+^ and slan^-^ ncMo was determined within the gate of ncMo.

**S2 Treatment with a non-blocking monoclonal antibody against CD16 (clone DJ130C) does not impact the haptokinetic response of slanMo.** Left: Images of the haptokinetic response of untreated slanMo and slanMo that were treated with a non-blocking monoclonal antibody against CD16 (clone DJ130C) Right: Percentage of untreated and treated polarized haptokinetic slanMo. N = 4. Error bars show mean ± SD.

**S3** **Haptokinesis is not induced by IgE ICs or R848.** Left: Images of the haptokinetic response of slanMo on IgG IC-coated, IgE IC-coated surfaces, with R848 prestimulation and PBS over a period of 15 min. Right: Percentage of polarized haptokinetic slanMo on IgG IC-coated and IgE IC-coated surfaces, after R848 treatment and on PBS. N = 4. Error bars show mean ± SD.

**S4 Toxicity assay.** % of PI positive cells after incubation with the SYK-inhibitor BAY61-3606. N = 3 Error bars show mean ± SD.

**S5 Antibodies deposited on endothelial cells do not induce a haptokinetic response in cMo.** Quantification of the haptokinetic response of cMo on antibody-targeted endothelial cells and non-targeted cells. Directionality is a measure of the straightness of cell migration. Each dot represents the mean of all tracked cells from one experiment. N = 5. Error bars show mean ± SD, **p < 0.01, ***p < 0.001, Kruskal Wallis test with Dunn’s post test.

**Supplementary Material and Methods**

**Haptokinesis assay on IgE ICs and with R848 stimulation.**IgE immuncomplexes were formed by incubation of human anti-NP IgE (absolute antibody, Redcar, UK) with NP-BSA (Biosearch Technologies, Hoddesdon, UK) for one hour at 4°C. The ICs were diluted to the same antigen concentration as the IgG ICs and slides were coated overnight at 4°C. For R848 stimulation of slanMo, slides were left untreated and cells were stimulated with 1 μg/ml R848 (Miltenyi Biotech) 15 min prior to acquisition. Haptokinesis assay was performed as described in the main Material and Method section of this manuscript.

**Haptokinesis assay on ICs with treatment with a non-blocking monoclonal antibody against CD16 (clone DJ130C).**Cells were pre-treated for 15 min at 4°C with a non-blocking monoclonal antibody against CD16 (clone DJ130C) at the same concentration (1 μg/ml) as used for sorting. After that, cells were washed and seeded on ICs. At the same time untreated slanMo from the same donor were seeded on ICs. Haptokinesis assay was performed as described in the main Material and Method section of this manuscript.

**Toxicity assay**
1x10^5^ slanMo in a 96 well plate were cultured in duplicates with indicated concentrations of the SYK inhibitor BAY61-3606 (Enzo LifeScience). After 3 hours, cells were harvested and stained with propidium iodide (BD, Heidelberg, Germany) directly before acquisition. Cells were analyzed using a Gallios flow cytometer (Beckman Coulter, Krefeld, Germany).
